# Supplementary material for: Identification of rare de novo epigenetic variations in congenital disorders
Source: Nat Commun. 2018 May 25;9:2064. doi: 10.1038/s41467-018-04540-x (PMC5970273; doi:10.1038/s41467-018-04540-x)
Supplement: Supplementary file 1 — Supplementary Information [file 41467_2018_4540_MOESM1_ESM.docx]

**Supplementary Information**

**“Identification of rare *de novo* epigenetic variations in congenital disorders” by Barbosa et al.**

| **Sample** | **Closest Gene** | **Imprinting** | **Chr_DMR** | **Start_hg19** | **End_hg19** | **Size** | **DMR** | **Bisulfite Seq** |
| --- | --- | --- | --- | --- | --- | --- | --- | --- |
| Proband398 | *MEG3* | Paternally methylated | chr14 | 101290194 | 101294429 | 4235 | hypo | True positive \| *De novo* |
| Proband146 | *MEG3* | Paternally methylated | chr14 | 101290194 | 101294429 | 4235 | hypo | True positive \| *De novo* |
| Proband308 | *L3MBTL1* | Maternally methyated | chr20 | 42142004 | 42143503 | 1499 | hypo | True positive \| *De novo* |
| Proband6 | *NAA60/ZNF597* | Paternally methylated | chr16 | 3493132 | 3494156 | 1024 | hypo | True positive \| Maternal |
| Proband62 | *NAA60/ZNF597* | Paternally methylated | chr16 | 3493132 | 3494156 | 1024 | hypo | Not tested |
| Proband130 | *JAKMIP1* | Maternally methyated | chr4 | 6107130 | 6107632 | 502 | hyper | True positive \| Suggestive of being *de novo* |
| Proband130 | *IGF1R* | Maternally methyated | chr15 | 99408635 | 99409956 | 1321 | hyper | True positive \| *De novo* |
| Proband92 | *SNU13* | Maternally methyated | chr22 | 42077938 | 42078722 | 784 | hypo | True positive \| *De novo* |
| Proband125 | *PPIEL* | Maternally methyated | chr1 | 40024970 | 40025414 | 444 | hypo | True positive \| Suggestive of being *de novo* |
| Proband421 | *IGF2R* | Maternally methyated | chr6 | 160426950 | 160427500 | 550 | hypo | True positive \| Suggestive of being *de novo* |
| Proband421 | *SNRPN* | Maternally methyated | chr15 | 25092065 | 25093523 | 1458 | hyper | Assay failed |
| Proband257 | *HM13* | Maternally methyated | chr20 | 30134928 | 30135361 | 433 | hyper | True positive \| Suggestive of being *de novo* |
| Proband164 | *HM13* | Maternally methyated | chr20 | 30134928 | 30135361 | 433 | hyper | True positive \| Suggestive of being *de novo* |

**Supplementary Table 1 | DMRs identified at imprinted loci in cases**

**
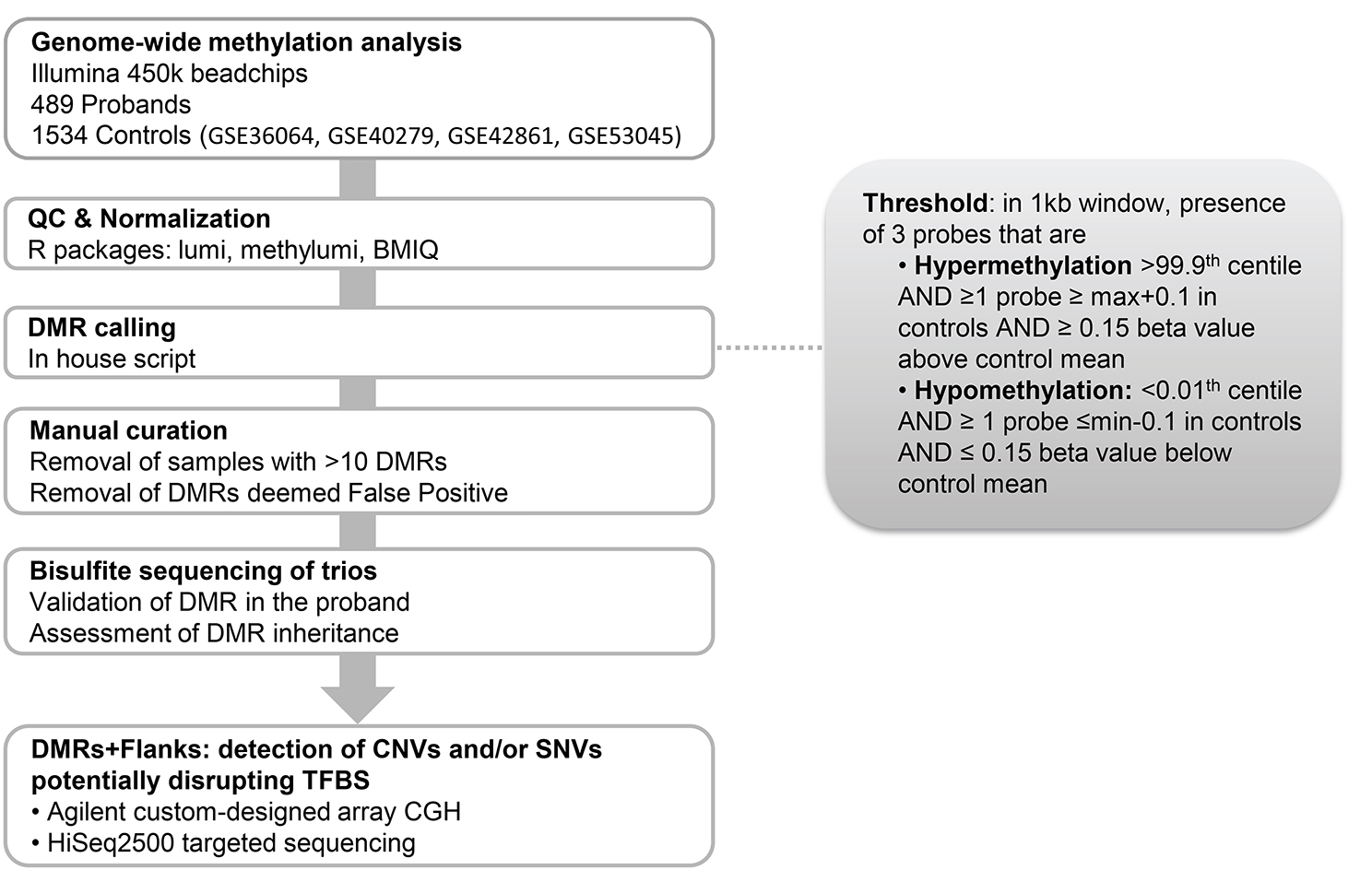
**

**Supplementary Figure 1 | Overview of the analysis pipeline.** Abbreviations: QC: Quality control: DMR: Differentially Methylated Region; CNVs: Copy Number Variants; Single Nucleotide Variants; TFBS: Transcription Factor Binding Sites.

**
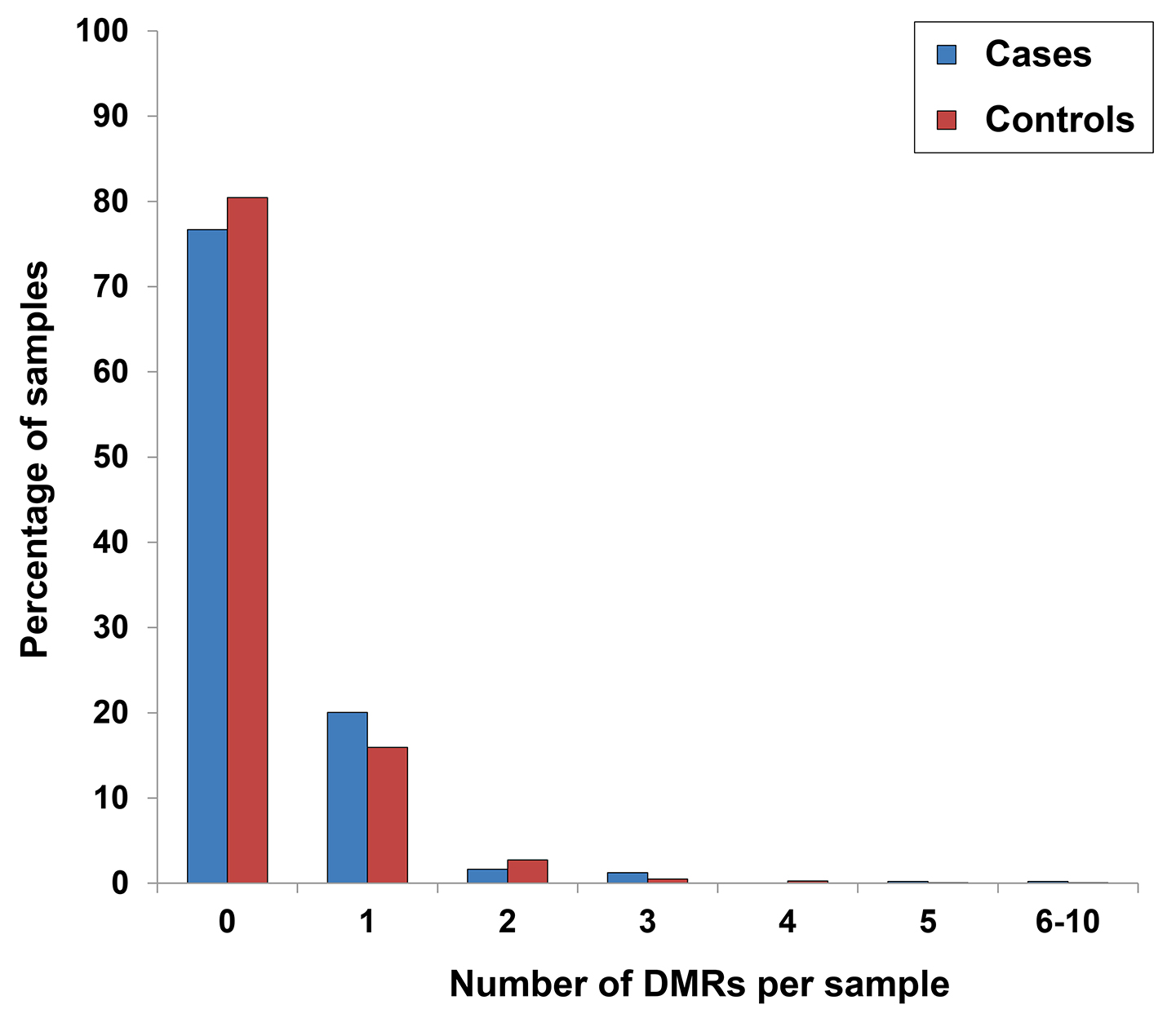
**

**Supplementary Figure 2 | Distribution of number of DMRs per sample - after QC.** DMRs kept after manual curation in 489 ND-CA cases (blue bars) and 2,711 unrelated population controls (red bars).


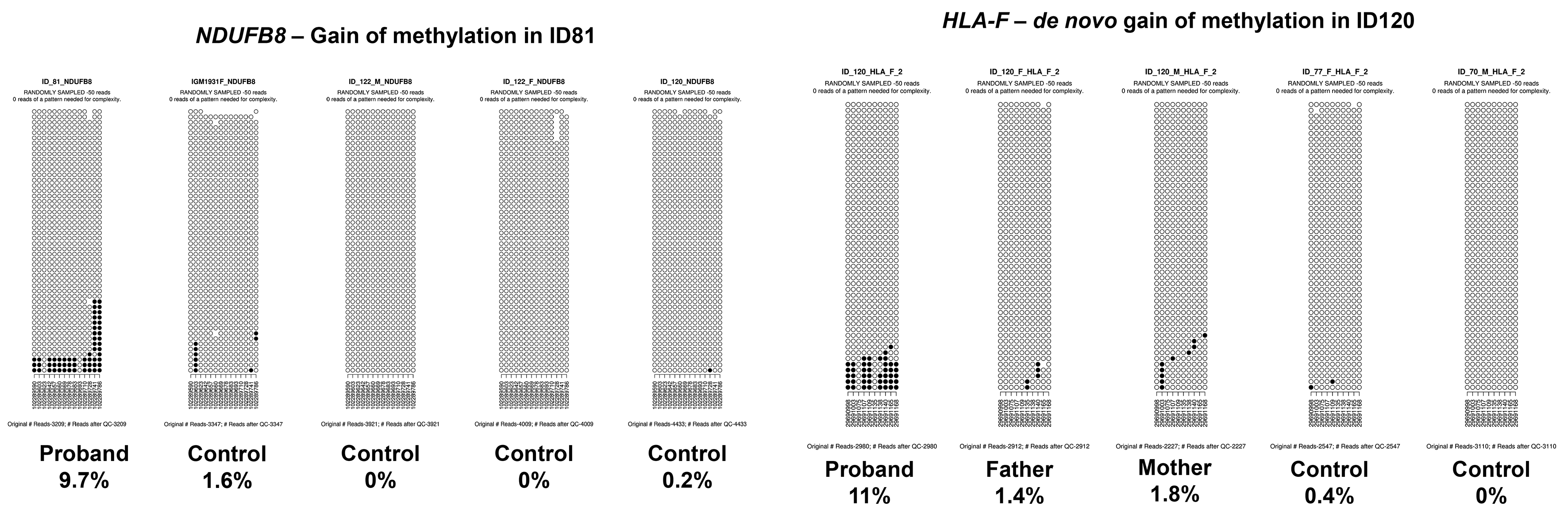


**Supplementary Figure 3 | Preferential amplification of the unmethylated allele complicates interpretation of PCR/bisulfite sequencing validation assays.** Using PCR/bisulfite sequencing, we attempted orthogonal confirmation for 70 epivariations. Overall, we scored 55 of the 58 assays that provided useful data as confirming the methylation changes detected by the 450k array (Supplementary Table 5). However, in some cases interpretation of these validation experiments was made complex due to highly biased allelic representation, presumably reflecting preferential PCR amplification of one allele. Both DMRs shown were sites where the array reported very low levels of methylation in controls, and a gain of methylation (*i.e.* hypermethylation) in the proband. Bisulfite PCR/sequencing results for both loci show that the proband possesses a population of highly methylated molecules (5-10% of the molecules sequenced), which was absent in all controls. As such, even though the percentage methylation change in proband versus controls for these amplicons was small, presumably due to the bisulfite PCR showing preferential amplification of the unmethylated allele, we considered these bisulfite PCR/sequencing assays to validate the array findings of a gain of methylation on one allele. Each plot 50 randomly selected molecules are shown, with each circle corresponding to a single CpG, and each row showing all CpGs tested in a single sequenced molecule: filled circles are methylated CpGs, open circles unmethylated CpGs.

**
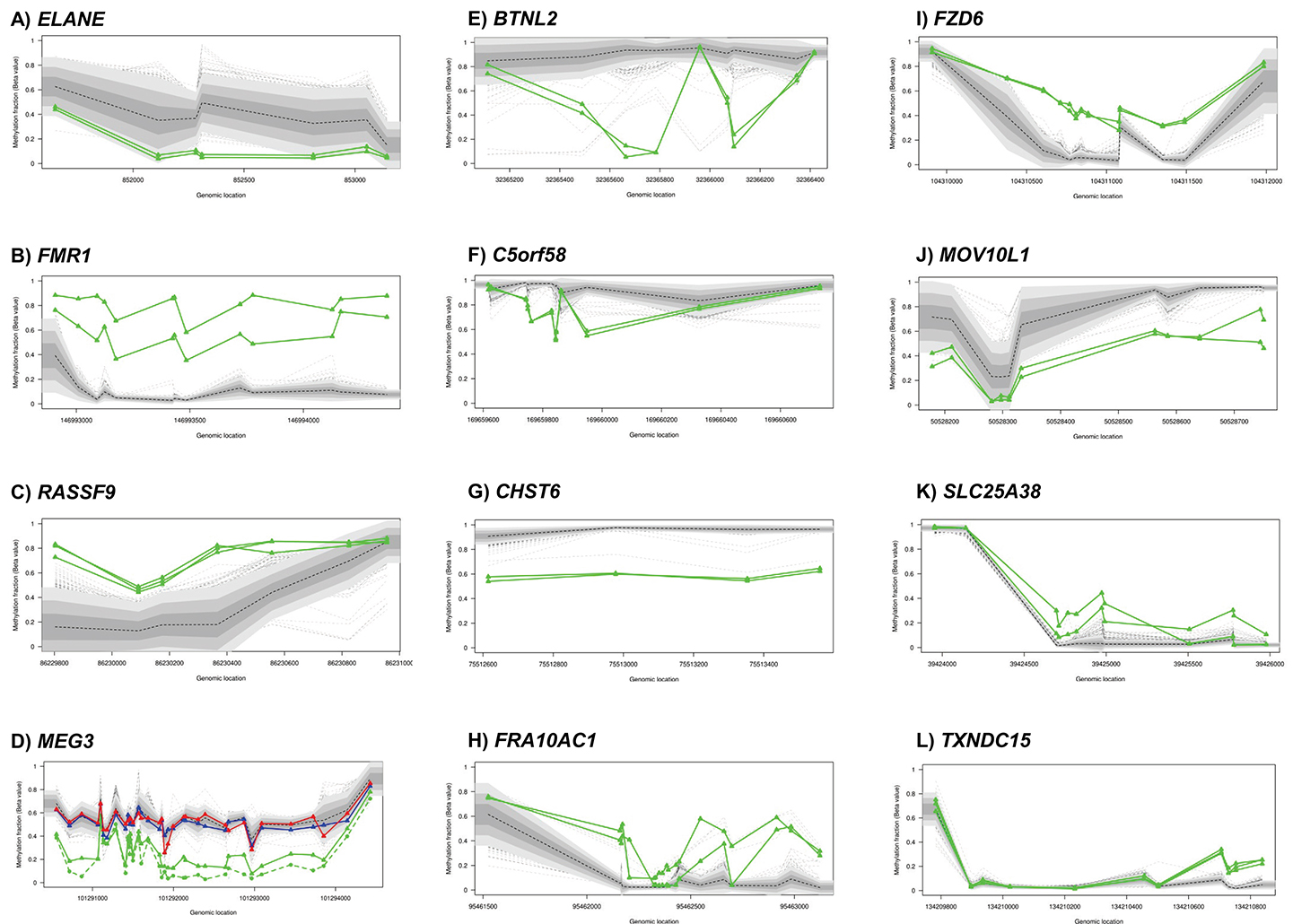
**

**Supplementary Figure 4 | Methylation plots of all recurrent epivariations.** The x-axis shows genomic coordinates (hg19), y-axis shows β value. **A)** *ELANE* (chr19:851649-853144) in Proband147 and Proband 9. **B)** *FMR1* (chrX:146992907-146994370) in Proband 311 and Proband 271. The β value in Proband 271 was around 0.6, suggestive of mosaicism. **C)** *RASSF9* (chr12:86229803-86230956) in Proband 269, Proband 298 and Proband 359. **D)** *MEG3* (chr14:101290194-101294429) in Proband 398 and Proband 146. **E)** *BTNL2* (chr6:32365112-32366416) in Proband 164 and Proband 22. **F)** *C5orf58* (chr5:169659617-169660733) in Proband 186 and Proband 193. **G)** *CHST6* (chr16:75512615-75513560) in Proband 353 and Proband 301. **H)** *FRA10AC1* (chr10:95461525-95463123) in Proband 308 and Proband 422. **I)** *FZD6* (chr8:104309909-104311984) in Proband 9 and Proband 127. **J)** *MOV10L1* (chr22:50528178-50528751) in Proband 22 and Proband 117. **K)** *SLC25A38* (chr3:39423953-39425977) in Proband 382 and Proband 129. **L)** *TXNDC15* (chr5:134209783-134210838) in Proband 451, Proband 293 and Proband 426. Five of these recurrent DMRs identified in our patient cohort were also observed in 2,711 population controls, (*C5orf58*, *CHST6*, *FRA10AC1*, *FZD6*), or in the 117 control pedigrees (*SLC25A38*).

**
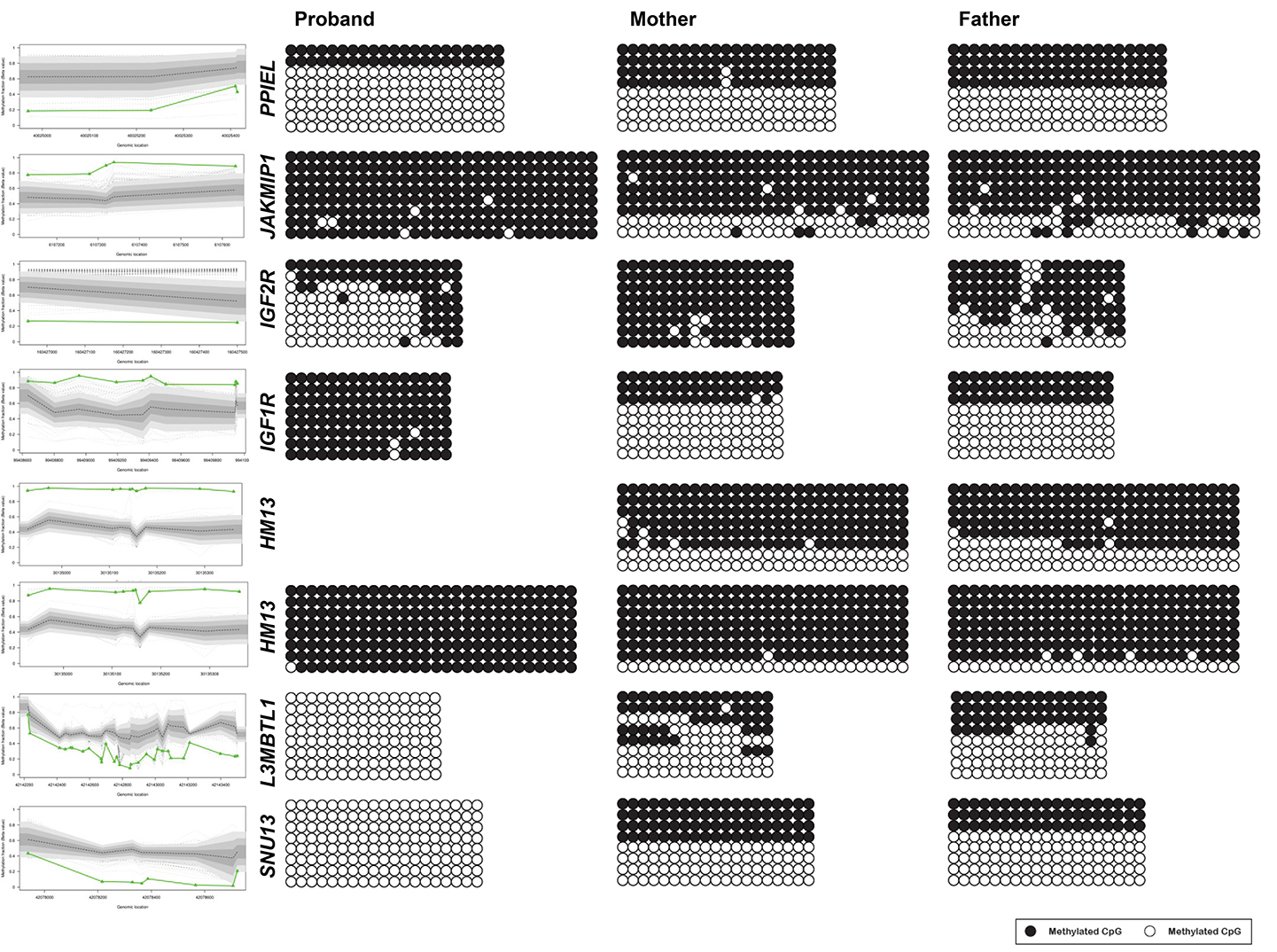
**

**Supplementary Figure 5 | Methylation plots and bisulfite sequencing of epivariations identified at known imprinted loci.** The x-axis shows genomic coordinates (hg19), y-axis shows β value. Outlier probands are highlighted in green. Bisulfite sequencing validated the Illumina 450k array findings and showed that the epivariations were *de novo* in Proband 130 (hypomethylation of *IGF1R* at chr15:99408635-99409956), Proband 308 (hypomethylation of *L3MBTL1* at chr20:42142004-42143503) and in Proband 92 (hypomethylation of *SNU13* at chr22:42077938-42078722). Results suggestive of the epivariations being *de novo* were observed in Proband 125 (hypomethylation of *PPIEL* at chr1:40024970-40025414), Proband 130 (hypermethylation of *JAKMIP1* at chr4:6107130-6107632), Proband 421 (hypomethylation of *IGF2R* at chr6:160426950-160427500), Proband 257 and Proband 164 (both with hypermethylation of *HM13* at chr20:30134928-30135361). Unfortunately there was insufficient DNA to perform bisulfite PCR for one proband with a gain of methylation at *HM13*, and thus we were unable to generate bisulfite sequencing data in this sample.


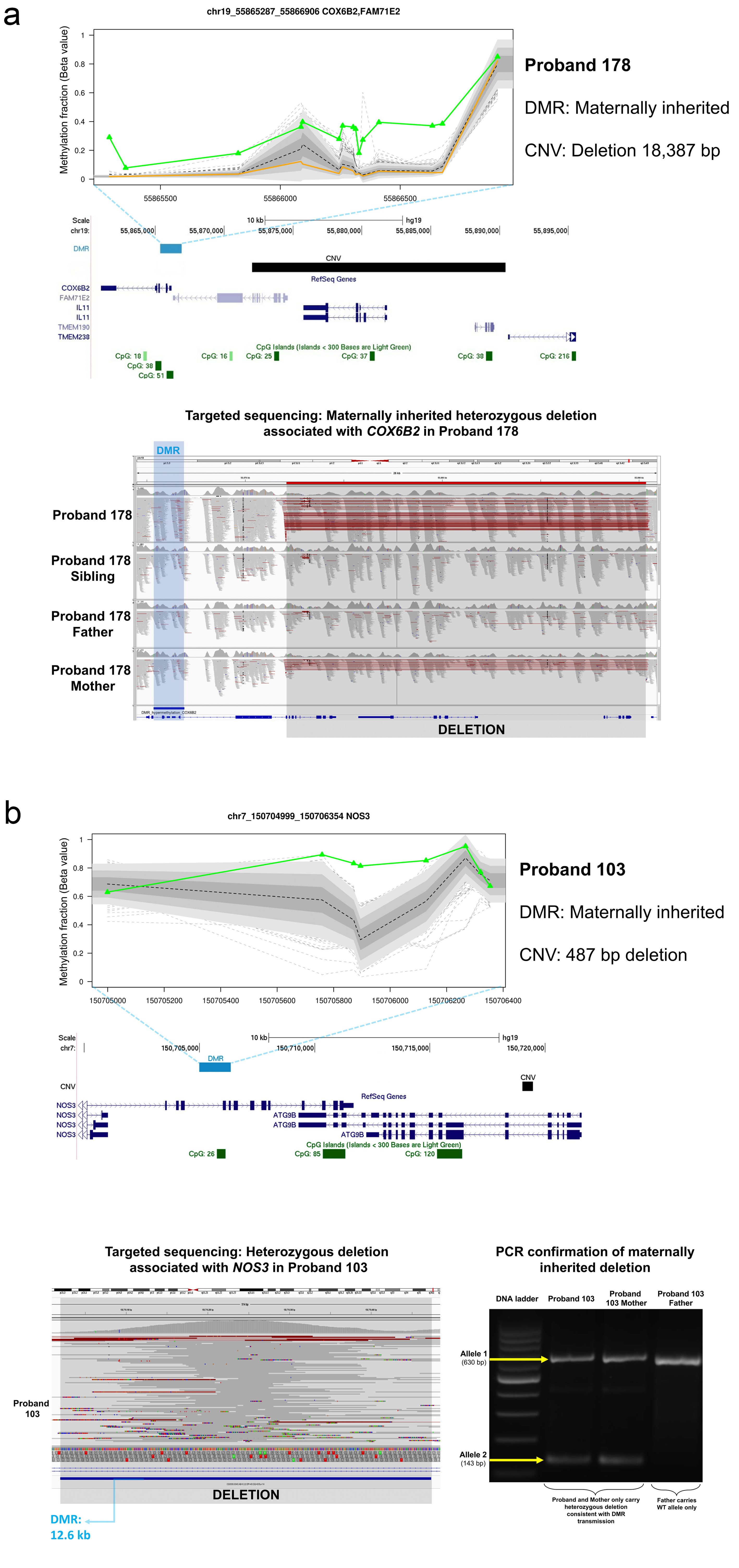


**Supplementary Figure 6 | Detection of rare CNVs by targeted sequencing of epivariations and their flanks. A)** Proband 178 carries a maternally inherited DMR at the *COX6B2* locus. We identified maternally inherited heterozygous 18,387 bp deletion downstream of the DMR**. B)** Proband 103 carries a maternally inherited DMR at the *NOS3* locus. We identified a maternally inherited heterozygous 487bp deletion located 13,204bp upstream of the DMR. Note that Proband103 was also found to carry an SNV within a CTCF binding site at this DMR (Figure 4).


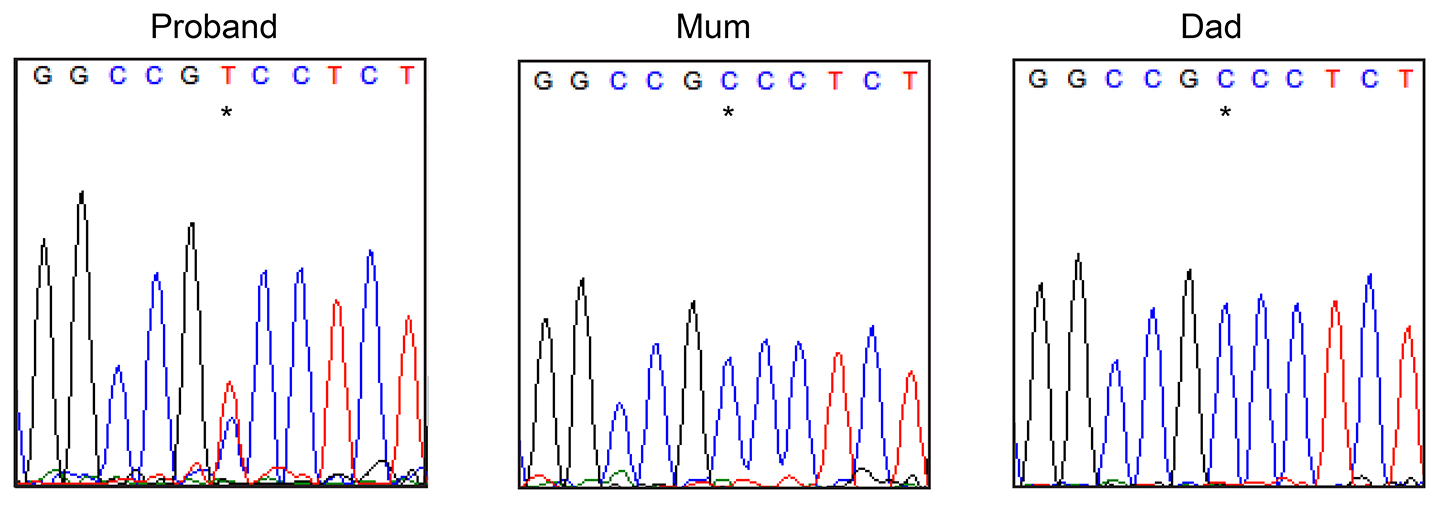


**Supplementary Figure 7 | Sanger validation of a *de novo* non-coding variant within a canonical CTCF binding motif associated with a *de novo* epivariation.** This rare *de novo* C>T SNV (hg19: chr19:295321) was initially detected by targeted sequencing, and lies within a *de novo* epivariation (hg19: chr19:294491-295738) identified in Proband 70. Given that the *de novo* SNV mutation rate is ~1 per 70 Mb^50^, the probability that a *de novo* SNV and a *de novo* epivariation would co-occur at the same locus by chance is very low. This strongly indicates a functional relationship between the two events, suggesting that this epivariation represents a secondary event caused by disruption of CTCF binding at the mutated motif.

**
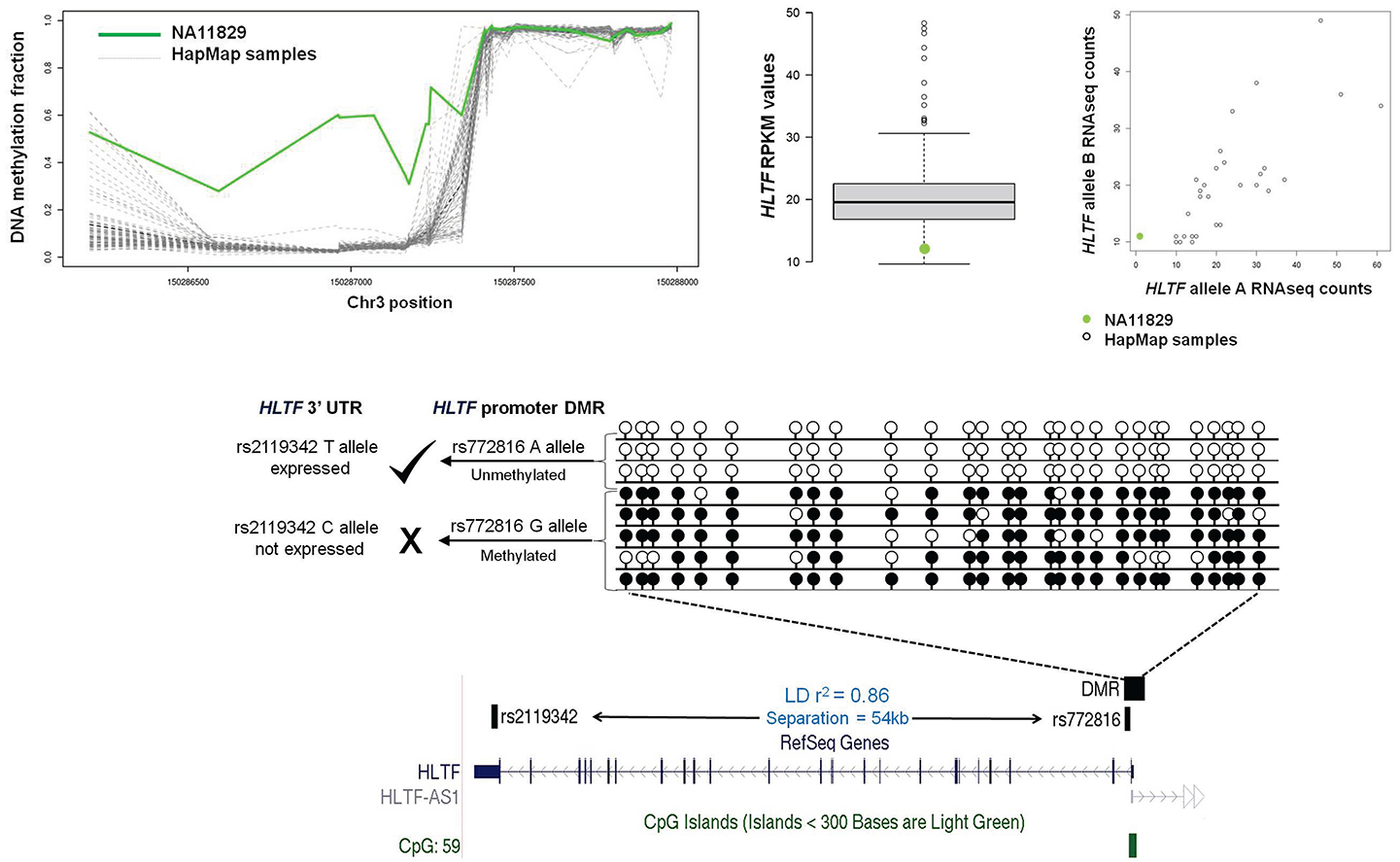
**

**Supplementary Figure 8 | Heterozygous promoter hypermethylation of *HLTF* is associated with allelic transcriptional silencing *in cis*.** A hypermethylated epivariation at the *HLTF* promoter was identified in NA11829. Using RNAseq data, NA11829 has unusually low and mono-allelic expression of *HLTF*. Allelic analysis of rs772816 shows a gain of methylation specifically on one allele. Phasing data shows the G allele of rs772816 lies *in cis* with the inactive 3’ UTR allele of rs2119342 used for allelic expression analysis. Thus, consistent with promoter hypermethylation causing transcriptional repression^22^, we observed that the methylated allele was located *in cis* with the transcriptionally silent allele. In the box plot, the center line shows the median; box limits indicate the 25^th^ and 75^th^ percentiles; whiskers extend 1.5 times the interquartile range from the 25^th^ and 75^th^ percentiles; outliers are shown as individual points.

**
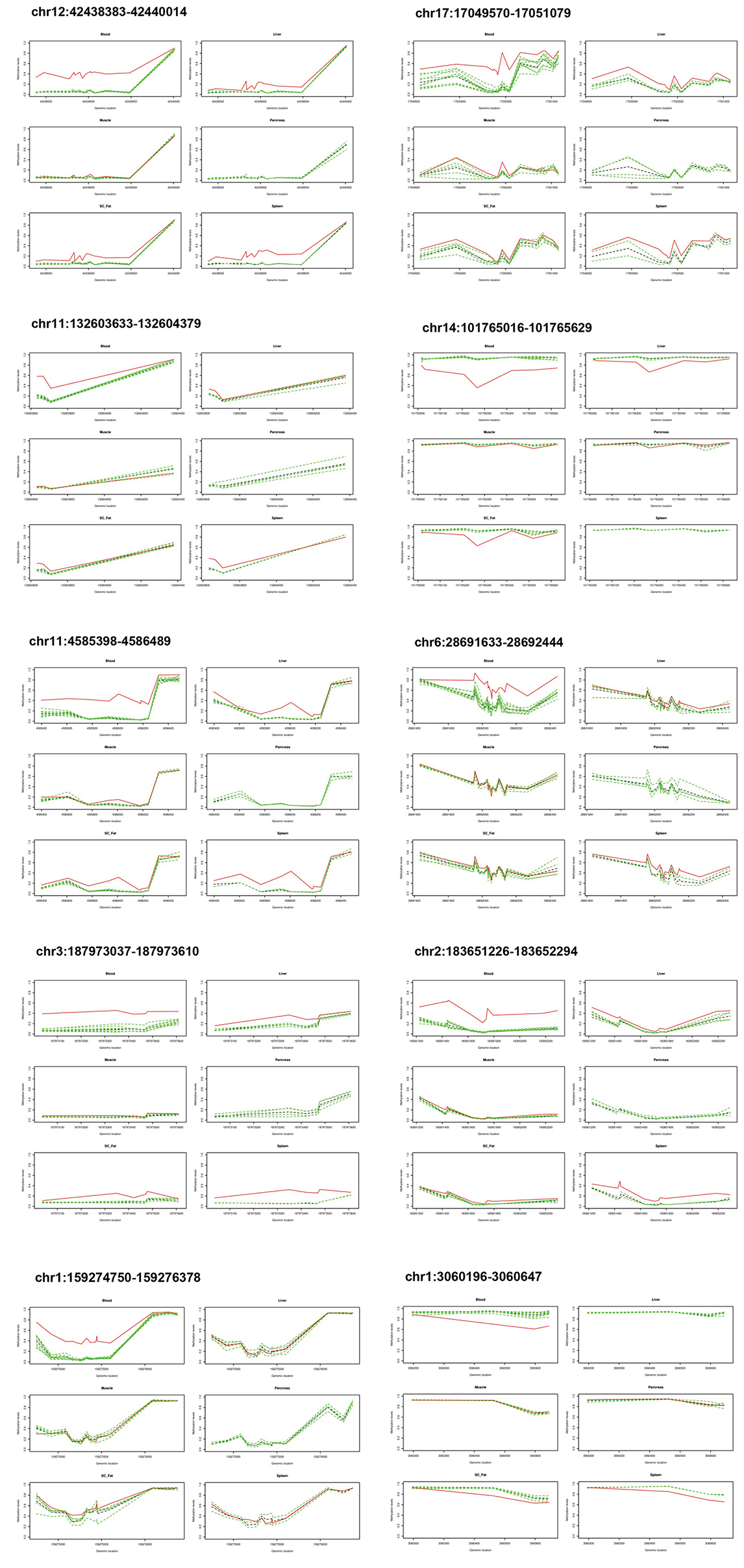
**

**Supplementary Figure 9 | Constitutive epivariations** **identified in six different tissues in controls.** Epivariations are generally constitutive events found across multiple different tissues within an individual. Using methylation profiles derived from six different tissues (Blood, Liver, Muscle, Pancreas, Fat and Spleen, GEO ID GSE48472) taken from cadavers^24^, we identified 10 epivariations in blood, and then studied these same loci in all other tissues available in the same individual. In every case, epivariations detected in blood also showed outlier methylation in other tissues isolated from the same individual.

**
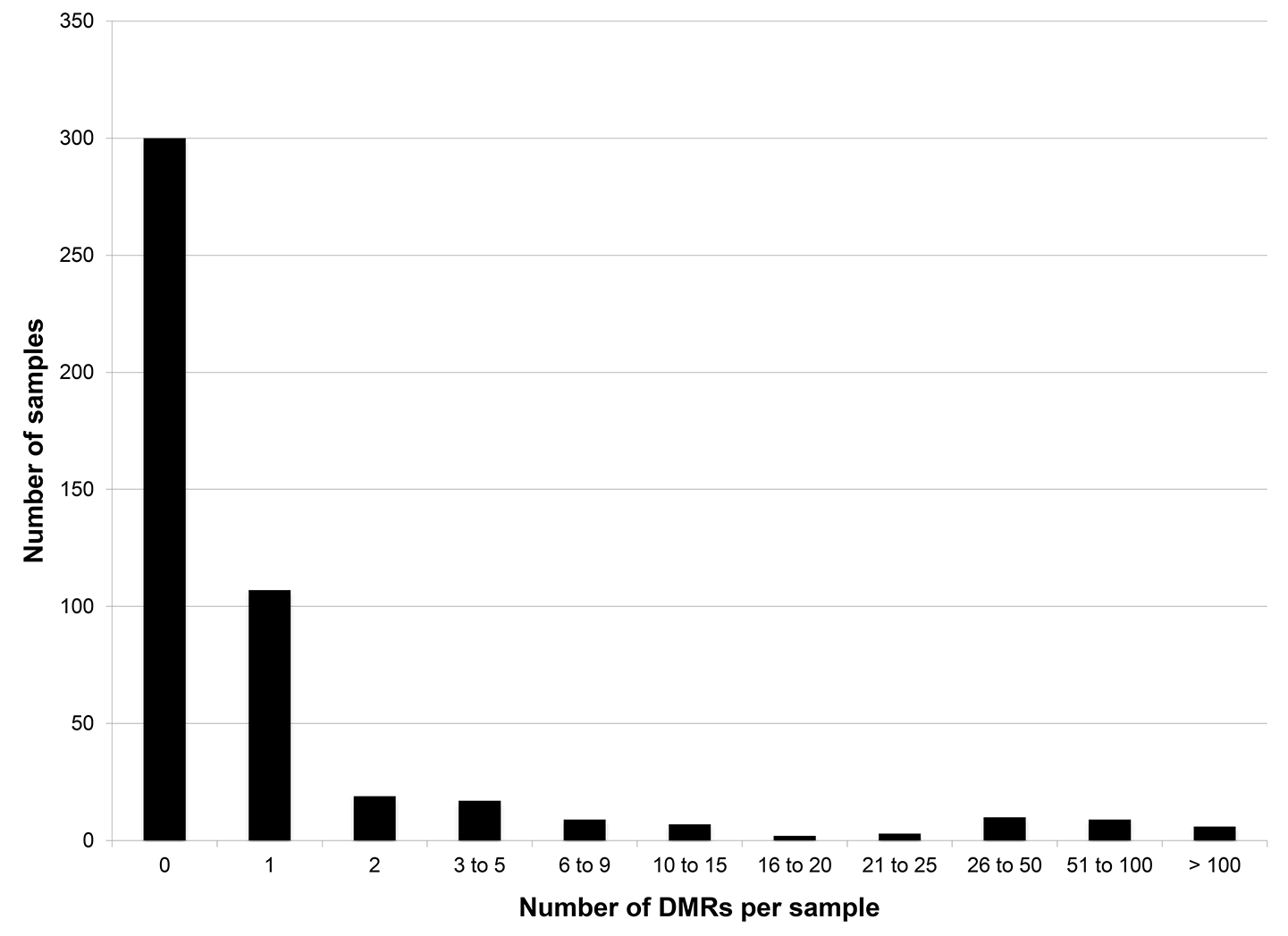
**

**Supplementary Figure 10 | Distribution of number of DMRs per sample - before QC**. All DMRs called by the custom perl script.
