# Supplementary material for: Identification of rare de novo epigenetic variations in congenital disorders
Source: Nat Commun. 2018 May 25;9:2064. doi: 10.1038/s41467-018-04540-x (PMC5970273; doi:10.1038/s41467-018-04540-x)
Supplement: Supplementary file 2 — Description of Additional Supplementary Files [file 41467_2018_4540_MOESM2_ESM.pdf]

Description of Additional Supplementary Files

File Name: Supplementary Data 1
Description: Clinical characterization of the cohort.

File Name: Supplementary Data 2
Description: DMRs identified in control families.

File Name: Supplementary Data 3
Description: DMRs identified in 2711 unrelated controls.

File Name: Supplementary Data 4
Description: DMRs identified in cases.

File Name: Supplementary Data 5
Description: DMR validation with bisulfite sequencing.

File Name: Supplementary Data 6
Description: CNVs detected with custom arrayCGH in cases.

File Name: Supplementary Data 7
Description: Rare SNVs within 75kb of DMRs in cases.

File Name: Supplementary Data 8
Description: Rare SNVs within 10kb of DMRs in 90 controls (1kGenomes).

File Name: Supplementary Data 9
Description: DMRs identified in multiple tissues in controls (Gencord).

File Name: Supplementary Data 10
Description: Gene expression and DMRs identified in 90 controls (1kGenomes).

File Name: Supplementary Data 11
Description: Putative homozygous deletions (clustered failed detection p values).
